# High-Intensity Interval Training and Sprint-Interval Training in National-Level Rowers

**DOI:** 10.3389/fphys.2021.803430

**Published:** 2021-12-14

**Authors:** Kirstie Jodie Turner, David Bruce Pyne, Julien D. Périard, Anthony John Rice

**Affiliations:** ^1^Research Institute for Sport and Exercise, University of Canberra, Canberra, ACT, Australia; ^2^Sports Science, Rowing Australia, Canberra, ACT, Australia

**Keywords:** rowing, training methodology, ergometer, performance, coaching

## Abstract

**Purpose:** The effects of two different high-intensity training methods on 2,000 m rowing ergometer performance were examined in a feasibility study of 24 national-level rowers aged 18–27 years (17 males, 2,000 m ergometer time trial 6:21.7 ± 0:14.6 (min:s) and seven females, 2,000 m ergometer 7:20.3 ± 0:12.1. Habitual training for all participants was ~12–16 h per week).

**Methods**: 16 high-intensity ergometer sessions were completed across two 3-week periods. Participants were allocated into two groups according to baseline 2,000 m time. High-intensity interval session-sprint-interval session (HIIT-SIT) completed eight HIIT (8 × 2.5 min intervals; 95% of 2,000 m wattage) followed by eight SIT (three sets of 7 × 30 s intervals; maximum effort). SIT-HIIT completed eight SIT sessions followed by eight HIIT sessions. Both a 2,000-m time trial and a progressive incremental test finishing with 4 min “all-out” performance were completed before and after each 3-week phase.

**Results:** Both groups showed similar improvements in 2,000 m time and 4 min “all-out” distance after the first 3 weeks (2,000 m time: HIIT-SIT: −2.0 ± 0.6%, mean ± 90% CL, *p* = 0.01; SIT-HIIT: −1.5 ± 0.3%, *p* = 0.01) with no significant difference between groups. HIIT-SIT demonstrated the greatest improvements in submaximal heart rate (HR) during the progressive incremental test with eight sessions of HIIT showing a greater reduction in submaximal HR than eight sessions of SIT. The net improvement of 16 high-intensity sessions on 2,000 m time was −2.5% for HIIT-SIT (−10.6 ± 3.9 s, *p* = 0.01) and − 2.2% for SIT-HIIT (−9.0 ± 5.7 s, *p* = 0.01) and for 4 min “all-out” performance was 3.1% for HIIT-SIT (36 ± 25 m, *p* = 0.01) and 2.8% for SIT-HIIT (33 ± 27 m, *p* = 0.01).

**Conclusion:** Eight sessions of high-intensity training can improve 2,000 m ergometer rowing performance in national-level rowers, with a further eight sessions producing minimal additional improvement. The method of high-intensity training appears less important than the dose.

## Introduction

A 2,000-m rowing race takes 5:30–8:00 min (depending on the racing category) and typically begins with a short supra-maximal start (~45 s), followed by 4–6 min of near maximal intensity, and finishes with another supra-maximal burst of 45–60 s. The race intensity distribution typically requires 70–75% of total energy from aerobic metabolism and 25–30% from anaerobic metabolism (Hagerman, 1984). Despite a substantial contribution from anaerobic metabolism, profiling of the training practices of elite rowers demonstrated that ~83% of training was undertaken at low intensities, 15–16% at or near anaerobic threshold and only 1–4% at high intensities (Tran et al., 2015). Current trends in cycling or running involve regular high-intensity, short-duration work bouts to drive physiological adaptation and performance improvement. The inclusion of high-intensity training, of both long and short work intervals, in addition to low-intensity endurance training purportedly yields superior improvements in endurance performance than low-intensity endurance training alone (Seiler, 2010; Stöggl and Sperlich, 2014).

Both high-intensity interval session (HIIT) and sprint-interval training (SIT) are increasingly common training methods used to stimulate adaptation in a range of endurance sports. Both training methods center on a reduction in training volume and increased training intensity to provide the stimulus for improved performance. The main difference between the training methods is the time domain of both the work and rest components, yielding a difference in the intensity distribution for each method. HIIT has been defined as a series of repeated short to moderate length intervals (up to 5 min in duration) completed at an intensity between the lactate threshold and maximal oxygen consumption (VO2max), separated by short and incomplete recovery periods, usually with a work:rest ratio ~ 1:1 (Laursen and Jenkins, 2002). HIIT primarily stimulates peripheral muscle metabolic changes in the short-term (Westgarth-Taylor et al., 1997; Weston et al., 1997; Burgomaster et al., 2005), while structural cardiovascular adaptations emerge over the long-term (Laursen et al., 2005; MacInnis and Gibala, 2017). Only three studies with varying intervention durations (4–8 weeks) have investigated the effect of HIIT on 2,000 m ergometer rowing performance. The improvements in performance ranged from 1.3 to 1.9% (5.0–8.2 s; Driller et al., 2009; Akca and Aras, 2015; Stevens et al., 2015; Ní Chéilleachair et al., 2017).

SIT and RST originated as a training method in team sports and selected endurance sports but is uncommon in rowing. RST is defined as three or more maximal short duration (≤30 s) efforts, interspersed with incomplete recovery periods (≤60 s) totaling up to ~15 min of sprint work (Millet et al., 2019). The terms SIT and RST are largely interchangeable in rowing and stimulate a high degree of neuromuscular and metabolic stress (Bishop et al., 2011), with the aerobic contribution increasing as a function of successive sprints (Bogdanis et al., 1996). In trained runners, RST improved 1,500 m time by 21 s (6%) after 7 weeks, despite a reduction in training volume of 50% (Gunnarsson and Bangsbo, 2012). Elite cyclists have shown improvements of 3.5–4.4% in a variety of key performance measures (e.g., 20 min self-paced time trial and peak aerobic power) after completing only nine RST sessions (Ronnestad et al., 2020).

To our knowledge, there are no published investigations that have directly compared the effects of short-term HIIT and SIT training in national- to elite-level rowers. Other sports, including cycling and running (Laursen et al., 2002; Gunnarsson and Bangsbo, 2012; Ronnestad et al., 2020), have been examined for this purpose, and we sought to extend this work to rowing. The aim of this study was to compare the effects of two successive training blocks of HIIT and SIT on 2,000 m ergometer rowing performance in national-level rowers. A secondary aim was to examine the time course of changes over the 16 sessions, irrespective of the training intervention, by comparing the two training blocks.

## Materials and Methods

### Participants

Thirty national- to elite-level rowers volunteered to participate in this study. A total of 24 rowers completed the entire study and were included in the final data analysis. Participants included 17 male rowers (age 22 ± 4 years; body mass 84.2 ± 12.7 kg; 2,000 m ergometer time 6:21.7 ± 0:14.6; mean ± SD) and seven female rowers (age 21 ± 1 years; body mass 66.7 ± 6.9 kg; 2,000 m ergometer time 7:20.3 ± 0:12.1) recruited from clubs and state institutes or academies of sport across Australia. The participants had competed at a national or international level in the previous season, consistently trained on-water 7–10 sessions per week. All participants were healthy, free from injury, and undertaking regular training sessions in adherence with COVID-19 guidelines prior to the study. Approval to conduct this study was provided by the University of Canberra Human Research Ethics Committee (approval 2020/444). All participants provided written informed consent after explanation of the aims, benefits, and risks of the study.

### Experimental Design

**Figure 1:**
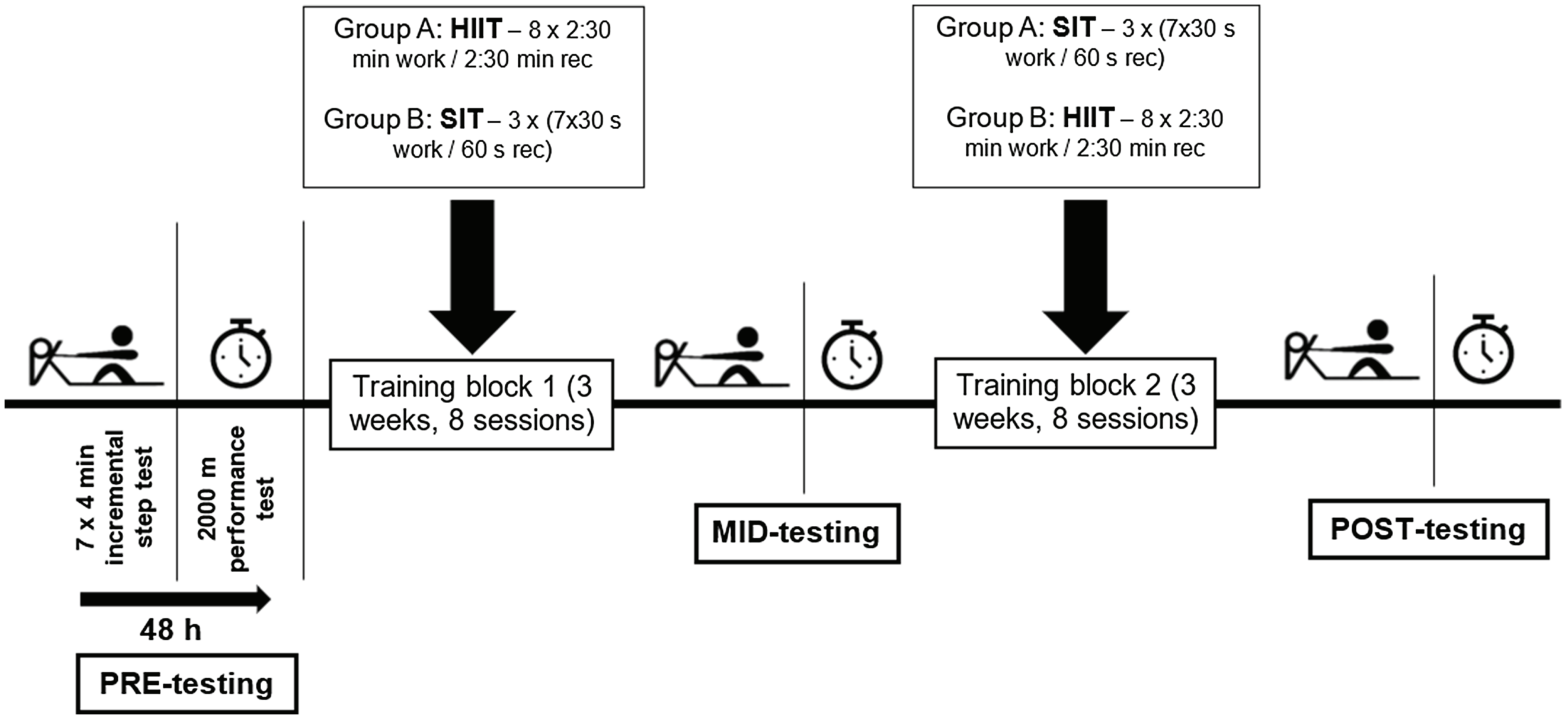
Experimental design showing PRE, MID, and POST-testing time points interspersed with training allocations according to GROUP.

A feasibility study was conducted to verify the likely effects of HIIT and SIT before a full randomized controlled trial study could be conducted (Whitehead et al., 2014). A longitudinal randomized cross-over design with two 3-week training conditions was employed to compare the effects of HIIT and SIT (Figure 1). Participants were informed that there was no clear advantage of one training type over the other. Participants performed baseline (PRE) testing and were then allocated randomly to either HIIT-SIT or SIT-HIIT, based on their 2,000 m performance test. Groups were counterbalanced in each training location to account for variability in training programs. HIIT-SIT completed 3 weeks of HIIT followed by 3 weeks of SIT, while SIT-HIIT completed 3 weeks of SIT followed by 3 weeks of HIIT. No additional high-intensity sessions were completed by rowers during this study to ensure all participants completed the same number of high-intensity workouts. All other training within each training location was prescribed by the coach and programmed to maintain within-subject consistency to standardize differences in training stimulus between the groups, or across the two training blocks. No control group was employed in the study as per previous recommendations for a feasibility study (Whitehead et al., 2014).

During each 3-week block, the participants completed a total of eight high-intensity training sessions of a single training method (HIIT or SIT) interspersed with their normal training (4–5 on-water, 3 strength, and 2–3 non ergometer cross-training). Training load (hours, % of time spent in training heart rate (HR) zones) was prescribed and remained stable for each individual across both 3-week training blocks. In a pilot study, the training load of a single HIIT and SIT session was determined, and the number of intervals and additional work prescribed to ensure each HIIT and SIT session created a standardized training stress for each individual. Training stress score was assessed using the T2-minute method (Tran et al., 2014), and pilot work facilitated modification of the high-intensity work bouts to ensure training stress score was matched across training styles. Following the first 3 weeks of training, participants had 1-week of light training (volume reduced by ~20%) during which they were re-tested (MID) and then assigned to the other training method. Following second 3-week training, block participants were re-tested (POST). All testing as well as HIIT and SIT training sessions were completed on a stationary Concept II rowing ergometer (Concept II Model D or E; Concept II Inc., Morrisville, United States). Minimum target workloads for HIIT in first 3-week intervention were based on the participant’s PRE 2,000 m mean power output. Target workloads for HIIT in second 3-week intervention were based on participant’s MID 2,000 m mean power output. All participants were provided with mobility, stretching, and muscular activation exercises to minimize the risk of overuse injury as a result of the introduction of high-intensity training sessions.

### Testing Protocol

Testing consisted of two sessions separated by 48 h and undertaken in accordance with COVID-19 restrictions, either in socially distanced groups or in a home environment. Day 1 of testing was undertaken 3 days following completion of the final high-intensity ergometer session, and day 2 of testing was undertaken a further 2 days later. Participants were familiar with the testing procedure and ergometer. The use of different ergometer models across the study was deemed acceptable given previous work showing testing on different models of the Concept II ergometer elicits near identical physiological responses (Vogler et al., 2007). The test–retest reliability of well-trained rowers on a Concept II ergometer is 0.7 ± 0.3% (mean ± 95% CL; Schabort et al., 1999).

On day 1 of testing participants completed a 7 × 4-min incremental ergometer step test (Rice and Osborne, 2013), with a 2,000-m ergometer time-trial undertaken 48 h later. To minimize diurnal variation of performance, each participant completed testing at approximately the same time of day throughout the study (Nugent et al., 2019), and on the same model ergometer. In the 24-h period prior to testing, the participants were instructed to avoid any strenuous activity, and consumption of alcohol. Before each testing session the ergometer drag factor was adjusted to correspond with the rower’s weight and gender category in accordance with Rowing Australia testing protocols (Rice and Osborne, 2013). These drag factors were 95 for lightweight women, 105 for lightweight men and heavyweight women, and 115 for heavyweight men.

### 7 × 4-min Incremental Step Test

Following a light warm-up, participants completed a 7 × 4-min incremental step test, interspersed with 1 min recovery periods. The starting workload and increments between workloads were individualized based on each participant’s best 2,000 m ergometer time in the previous 12 months (Rice and Osborne, 2013). Workloads 1–6 were submaximal and workload 7 was a maximal effort, acting as a 4-min time-trial (4minTT). Each participant was required to hold their prescribed power outputs for workload 1–6, and then instructed to cover as much distance as possible during workload 7. Power output, stroke rate, and HR were recorded on the ergometer work monitor and averaged during each of the 4 min workload periods. The rating of perceived exertion (RPE) was recorded during the recovery period using the Borg 6–20 scale (Borg, 1970). Distance covered in the final 4 min and mean power output were recorded as the criterion dependent variables.

### 2,000 m Performance Test

Participants undertook stretching and a self-selected 30 min warm-up prior to each 2,000 m testing session. The display module on the ergometer was set to record the mean power output and stroke rate for each 100 m increment. HR was recorded continuously throughout the test (Wahoo Tickr; Wahoo Fitness, Georgia, United States) and RPE recorded immediately upon completion. Max HR was reported as the maximum HR recorded during either test. Time to complete 2,000 m and mean power output were recorded as the criterion dependent variables.

### Training Intervention Protocols

HIIT or SIT sessions were completed three times per week for 3 weeks, yielding eight sessions in total (only two sessions were completed in the final week to allow for additional recovery before testing the following week). Each intervention session replaced a normal endurance training session in the program. Power output, HR, and RPE were recorded for each set of each session. In addition to the training intervention sessions, training consisted of 4–5 endurance sessions, 3 strength sessions, and 2–3 cross training sessions per week. In some training locations, on-water was not possible due to local COVID-19 restrictions; therefore, cycling and running were substituted for in for on-water sessions. The ergometer monitor was set to display the work and rest interval duration. Both sessions lasted ~60 min.

### High-Intensity Interval Training

Each HIIT session consisted of a 10-min self-selected warm-up, repeated before every session, followed by eight intervals at ≥95% of 2,000 m mean power output. Each interval was 2.5 min in duration separated by a 2.5-min recovery period (Millet et al., 2019). Participants were instructed to try and improve their mean power output each session. After the completion of each work interval, HR and RPE data were recorded with an extra 2.5 min break following interval 4.

### Sprint-Interval Training

Each SIT session consisted of a 10-min self-selected warm-up, repeated before every session, followed by three sets of seven “all-out” (~130% of 2,000 m mean power output) intervals using full length strokes. Each interval was 30 s in duration and interspersed with 60 s of recovery (Laursen et al., 2002). The stroke rate was capped at 40 strokes.min^−1^ to ensure technically sound strokes were completed. There was a 5-min recovery period after each set, where RPE data were recorded.

### Statistical Analysis

Mean, standard deviation (SD), and 90% confidence limits (CLs) were calculated for each testing and training variable. Percentage of maximum heart rate (%HRMAX) was calculated from each participant’s highest value achieved in either of the two PRE performance trials. Statistical Package for Social Sciences (SPSS) software (Version 25, SPSS Inc., Illinois, United States) was used for statistical analyses. Linear mixed modeling was employed to determine differences in training method (HIIT and SIT) across the two 3-week interventions. The fixed effects factor was HIIT vs. SIT, and the random effects were the change in the dependent variables (2,000 m time and 4minTT power output) over time. The first testing block was analyzed independently to assess the rowing performance effects of SIT and HIIT following a single block of a specific training method, with a Bonferroni correction employed for all *post hoc* analyses. Sample size estimation using G*Power software (v3.1.9.4 *a priori* power analysis with ANOVA repeated measures, within-between interaction, *α* = 0.05, 1 − *β* = 0.80, Cohen’s *d* = 0.2) indicated the study required 24 participants (Faul et al., 2007). An additional six participants were added to account for an assumed 25% dropout rate. Statistical significance was set at *p* < 0.05.

## Results

Of the 24 participants who completed the study, HIIT-SIT was comprised *n* = 11 and SIT-HIIT was comprised *n* = 13 participants (Table 1).

**Table 1:** Mean body mass and 2,000 m rowing ergometer PB for heavyweight and lightweight athletes in HIIT-SIT and SIT-HIIT groups.

Variable	Group	Heavyweight men	Lightweight men	Heavyweight women	Lightweight women
Body mass (kg)	HIIT-SIT	90.8 ± 9.9	73.9 ± 0.2	75.5 ± 5.7	60.8 ± 0.2
	SIT-HIIT	97.8 ± 9.5	73.1 ± 2.1	67.7 ± 1.2	60.9 ± 0.0
2,000 m PB (s)	HIIT-SIT	375.2 ± 16.1	381.1 ± 7.6	433.9 ± 26.6	444.7 ± 0.4
	SIT-HIIT	373.5 ± 9.9	392.8 ± 12.6	444.2 ± 1.3	436.7 ± 0.0

*Data are represented as mean ± SD*.

### Two Blocks of High-Intensity Training

Table 2 displays changes in performance measures for the 2,000 m and 4minTT rowing tests. When compared to baseline, both HIIT-SIT and SIT-HIIT improved (*p* = 0.01) both 2,000 m and 4minTT performance (power output, distance covered in 4 min, and time to complete 2,000 m) after 9 weeks of the study. However, there was no significant difference in rowing performance between the training methods (*p* = 0.62). Changes in performance following the first 3-week training intervention for both HIIT-SIT and SIT-HIIT were significantly different to PRE values, but performance was not further improved following the second 3-week intervention, where the order of the training methods was reversed.

**Table 2:** HIIT-SIT and SIT-HIIT results for 2,000 m rowing ergometer performance (2,000 m) and incremental step test peak performance (4minTT) for PRE, MID, and POST.

Variable	Group	PRE	MID	POST	Δ PRE to MID	Δ MID to POST	Δ Overall
2,000 m finish time (s)	HIIT-SIT	411.5 ± 36.5	403.1 ± 35.0	401.0 ± 34.8	−8.4 ± 5.2*	−2.1 ± 5.5	−10.6 ± 7.8^*^
	SIT-HIIT	407.5 ± 28.2	401.5 ± 28.0	398.6 ± 28.0	−6.0 ± 3.1^*^	−2.9 ± 4.4	−9.0 ± 5.7^*^
2,000 m PO (W)	HIIT-SIT	334 ± 82	356 ± 86	361 ± 87	21 ± 13^*^	5 ± 15	27 ± 20^*^
	SIT-HIIT	339 ± 65	355 ± 68	363 ± 72	16 ± 8^*^	8 ± 11^*^	24 ± 15^*^
4 min TT distance (m)	HIIT-SIT	1,162 ± 117	1,188 ± 108	1,198 ± 109	26 ± 17^*^	10 ± 18	36 ± 25^*^
	SIT-HIIT	1,177 ± 80	1,197 ± 77	1,210 ± 84	20 ± 18	13 ± 23^*^	33 ± 27^*^
4 min TT PO (W)	HIIT-SIT	329 ± 90	353 ± 90	362 ± 93	24 ± 15^*^	9 ± 14	33 ± 21^*^
	SIT-HIIT	333 ± 67	348 ± 65	360 ± 73	14 ± 12	12 ± 22^*^	27 ± 23^*^

*PRE, MID, and POST data are represented as mean ± SD. Change data (Δ) are represented as mean ± 90% CL*.

* *Significantly different from PRE (p < 0.05)*.

### Eight Sessions of High-Intensity Training: HIIT Vs. SIT

**Figure 2:**
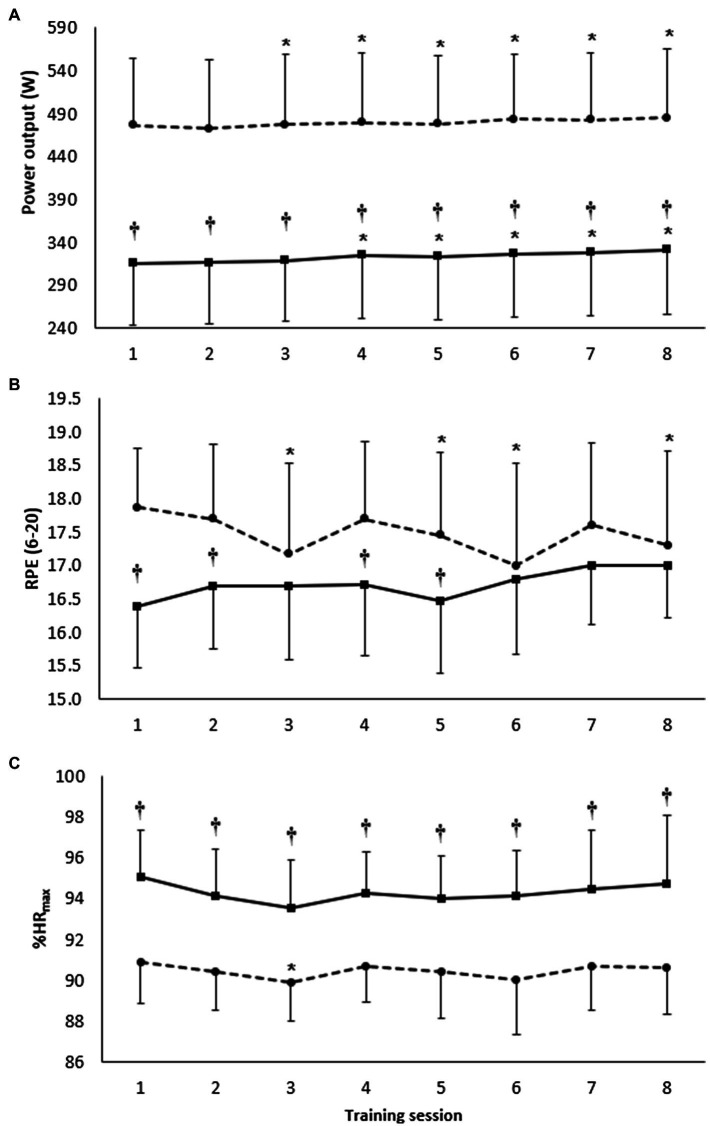
Panel **A** shows mean power output (W), panel **B** shows rating of perceived exertion (RPE 6–20), and panel **C** shows heart rate as a percentage of maximum (%HRmax) for BLOCK1 training sessions. HIIT is represented by the solid line and SIT is represented by the dashed line. Data are displayed as mean ± SD. *Significantly different to session 1 (*p* < 0.05). †Significant between HIIT and SIT (*p* < 0.05).

When data for the first 3-week intervention was analyzed as a single training block, both HIIT and SIT improved 2,000 m and 4minTT (Table 2). Eight sessions of HIIT resulted in a −2.0± 0.6% (mean ± 90%CL; *p* = 0.01) improvement in 2,000 m time and 2.6 ± 0.9% (*p* = 0.01) improvement in 4minTT distance. Similarly, eight sessions of SIT resulted in −1.5 ± 0.3% (*p* = 0.01) improvement in 2,000 m time, and 1.5 ± 0.3% (*p* = 0.06) improvement in 4minTT distance. There were no significant differences in either performance test when HIIT and SIT were compared for the first 3-week intervention (*p* = 0.68). Both HIIT and SIT training methods elicited improvements in power output of 10–15 W from the first to the eighth training session (Figure 2A).

HIIT sessions were associated with a lower mean session power output and higher %HRmax across all eight training sessions when compared with SIT (Figures 2A,C, respectively, *p* < 0.05). RPE was consistently lower during HIIT sessions, but only different from SIT for sessions 1, 2, 4, and 5 (Figures 2B, *p* < 0.05). Relative to session 1 of the corresponding training method, sessions 4–8 were completed with a greater power output for HIIT (*p* < 0.05), while sessions 3–8 were completed with a greater power output for SIT (*p* < 0.05).

### Comparison of Consecutive Training Blocks

Table 3 shows the change in performance for 2,000 m and 4minTT measures when data were collapsed and analyzed for the first 3-week intervention vs. the second 3-week intervention, irrespective of the training intervention (HIIT or SIT). The greatest improvements in both 2,000 m and 4minTT were realized following the initial 3-week intervention (*p* = 0.01), with no further improvements occurring following another 3 weeks of training (*p* = 0.75).

**Table 3:** Change in performance measures following the first training block (BLOCK1) and the second training block (BLOCK2).

Variable	First 3-week block	Second 3-week block	Both 3-week blocks
Δ 2,000 m time (%)	−1.7 ± 0.3^*^	−0.6 ± 0.4^†^	−2.3 ± 0.5^*^
Δ 2,000 m power output (%)	5.5 ± 1.1^*^	2.0 ± 1.2^†^	7.6 ± 1.7^*^
Δ 4 minTT distance (%)	2.0 ± 0.5^*^	1.0 ± 0.6^†^	3.0 ± 0.8^*^
Δ 4 minTT power output (%)	6.2 ± 1.7^*^	3.1 ± 1.9^†^	9.4 ± 2.5^*^

Values are shown as mean percentage change ±90% CL.

* *Significantly different to PRE (p < 0.05)*.

† *Significant between first and second 3-week blocks (p < 0.05)*.

### Submaximal Performance

HIIT-SIT elicited marked reductions in HR at STEPs 4, 5, and 6 after 16 sessions (*p* = 0.02). In contrast, in SIT-HIIT only HR at STEP 6 of POST was reduced (*p* = 0.07). HIIT-SIT yielded a lower HR compared to SIT-HIIT (*p* < 0.05) after eight and 16 sessions. RPE was similar for both groups throughout the training interventions.

## Discussion

The major outcomes of this feasibility study were that both HIIT and SIT improved rowing performance (2,000 m time: 9.0–10.6 s and 4minTT power output: 27–33 W) after 16 sessions (Table 2). However, there was no significant difference in the magnitude of the improvements between HIIT and SIT training. Given this lack of difference between HIIT and SIT for any performance variable after both eight and 16 sessions, it appears both methods of training are viable options for coaches looking for short-term improvements in performance in highly trained rowers. The magnitude of performance improvement over 2,000 m (~10 s) compares very favorably with changes in performance following extended endurance training. This study is the first to demonstrate successive blocks of HIIT and SIT can improve rowing performance in national to elite-level rowers, with performance changes seemingly more dependent on the inclusion of high-intensity training (HIIT or SIT), than the specific nature of the intervals.

Investigations of HIIT in rowers have been largely confined to three studies. Driller et al. (2009) completed a randomized cross-over design with 4 weeks of HIIT and control training balanced across two matched groups. Akca and Aras (2015) implemented a 4-week block of HIIT. Ní Chéilleachair et al. (2017) performed a straight 8-week block of high-intensity training. Regardless of study design and the work:rest ratio employed; these investigations demonstrated that HIIT improves rowing performance to a similar degree (1.3–1.8%). In the current study, our analysis of a single training block demonstrated that HIIT and SIT yielded similar improvement (1.5–2.0%) after only eight sessions across 3 weeks. This outcome indicates that substantial improvements in performance can be realized in a shorter block of time than previous investigations. Importantly, a 3-week HIIT or SIT mesocycle is more viable for coaches to implement into a seasonal program than a longer 8-week block.

The first 3-week training intervention induced the greatest change in rowing performance regardless of training method (HIIT or SIT) with an additional 3-weeks of training yielding no further significant improvement in rowing performance. By session 4, both HIIT and SIT had already elicited higher average power output during the work intervals when compared with the first session of the respective training method. After the fifth session, there were no further substantial increases in power output for either training method. Our data indicate at least five high-intensity sessions may be required to induce a substantial change in rowing performance, and that beyond eight sessions there appears to be little additional improvement in rowing performance. Individual responses in the second 3-week block of training showed greater variation, with most rowers in both groups responding with either no further improvement, or a trivial improvement, in performance. Irrespective of the type of high-intensity training undertaken in the second block of training, athletes did not show deteriorations in performance. It is interesting to speculate on how best to periodize the inclusion of high intensity training in a typical competitive season. Our results appear to indicate that appropriate periodization of eight sessions of high-intensity training (HIIT or SIT) periodized once every three macrocycles (one macro cycle = 4 weeks) could promote incremental improvements in 2,000 m performance.

In this investigation the first 3-week training block was used to examine the performance benefits of eight sessions of HIIT compared with SIT when all other training was matched between groups at each training location. Our data showed that 2,000 m time improved for both HIIT-SIT and SIT-HIIT by 2.0 and 1.5%, respectively, (Table 3). Three other studies have been conducted in rowers where a variety of training methods were compared in a single training cycle (i.e., 4 weeks or 8–10 sessions). These investigations have yielded improvements in 2,000 m time of 5.7 s (1.4%), 5 s (1.2%), and 4 s (1%) with HIIT (2.5 min work with 3 min active recovery), supramaximal interval training (10 × 30 s max with 4 min active recovery), and SIT (4–6 × 60 s max with 2.5–5 min recovery), respectively (Driller et al., 2009; Akca and Aras, 2015; Stevens et al., 2015). These data are consistent with those from the present study and support the principle of training specificity (Hewson and Hopkins, 1996), confirming the notion that inclusion of HIIT, or SIT can improve 2,000 m rowing performance in as few as eight sessions in national-level rowers.

The submaximal responses to high-intensity training in rowing have largely been unreported in previous studies. While maximal performance benefits are often the primary indicator of success of a training intervention, such benefits are only realized after training induces the appropriate improvement in submaximal efficiency. While we were unable to complete a more in-depth metabolic analysis of our training due to COVID-19 restrictions, the results from the submaximal steps from our 7 × 4 testing protocol indicated HIIT-SIT reduced HR during STEP 6 after 16 sessions of high-intensity training. After only eight sessions HIIT-SIT showed a greater reduction in HR at STEP 4, 5, and 6 than SIT-HIIT. The combination of HIIT followed by SIT in the present investigation resulted in the greatest improvement in submaximal HR. Investigations in other sports (alpine skiers and soccer players) have also reported similar reductions in submaximal HR at a given workload following high-intensity training (Briel et al., 2010; Faude et al., 2014). Further research using a more detailed physiological assessment of the submaximal responses to HIIT and SIT is warranted. The next step will include a full randomized controlled trial to extend on the work of this study (Whitehead et al., 2014).

The sole outcome of this investigation was to determine the effect of concentrated blocks of high intensity training on maximal rowing performance. In achieving this outcome, it is worthy to consider the physiological and biochemical mechanisms underpinning the clear improvements in 2,000 m and 4 min “all-out” performances. Previous investigations utilizing work:rest and intensity domains similar to those used in this investigation reported changes in both intracellular and extracellular buffering capacity, heart remodeling and cardiac function, skeletal muscle oxidative capacity, submaximal oxygen efficiency, and the central nervous system (Weston et al., 1997; Shigenori, 2019; Callahan et al., 2021; Hu et al., 2021). Although we have no specific data to add to the underlying mechanisms, it appears that given the high baseline training status of the participants, coupled with the relatively short exposure time to the high intensity training regime, the major alteration in physiology and biochemistry that led to the positive changes in maximal rowing performances were biochemical rather than structural (i.e., heart remodeling) in nature. Rapid improvements in intra- and extra-cellular buffering capacities are likely responsible for the measured changes in maximal rowing performances reported in this study. Mechanistic studies typically involve moderately active participants rather than elite athletes, and further work in this area is required to provide greater understanding of mechanisms underpinning performance improvements with HIIT and SIT.

This study was conducted during the height of the COVID-19 pandemic. As a direct result we chose not to include a control group as the HIIT and SIT protocols were used as training motivation for athletes while conforming to local “social distancing” guidelines. This limitation has implications in quantifying the true effects of HIIT or SIT by accounting for the control group response. A similar protocol involving more traditional endurance training over 4 weeks induced improvements of 0.4–0.5% (Driller et al., 2009; Stevens et al., 2015). This limitation should be considered when evaluating the responses to HIIT or SIT in the present study, and future work could extend this using a more traditional randomized controlled trial design.

## Practical Applications

It appears that as few as eight sessions of high-intensity training (HIIT or SIT) can substantially improve rowing ergometer performance in elite rowers, and both HIIT and SIT are viable training options for coaches. Based on self-reported perceptual feedback, HIIT was better tolerated by the athletes and as such may be more favorable if only one style of high intensity training can be employed. Undertaking a second block of high-intensity training should be considered by coaches on a case-by-case basis, given the marked individual variability in responses.

## Conclusion

A total of eight sessions of HIIT or SIT substantially improved 2,000 m rowing ergometer performance; however, 4minTT performance only improved after HIIT. There was no marked difference in the performance improvement between the training interventions. The greatest improvement in performance occurred following the first training block, and a second 3-week training block of HIIT or SIT did not result in further improvements in either 2,000 m or 4minTT performance. When selecting a training intervention, HIIT should be considered for an 8-session block while there is no difference between training styles across 16 sessions.

## Data Availability Statement

The original contributions presented in the study are included in the article/supplementary material; further inquiries can be directed to the corresponding author.

## Ethics Statement

The studies involving human participants were reviewed and approved by the University of Canberra Human Research Ethics Committee. The patients/participants provided their written informed consent to participate in this study.

## Author Contributions

KT and AR were responsible for all aspects of this manuscript. DP and JP were responsible for the development of the study protocol, data analysis, and manuscript revisions. All authors contributed to the article and approved the submitted version.

## Conflict of Interest

The authors declare that the research was conducted in the absence of any commercial or financial relationships that could be construed as a potential conflict of interest.

## Publisher’s Note

All claims expressed in this article are solely those of the authors and do not necessarily represent those of their affiliated organizations, or those of the publisher, the editors and the reviewers. Any product that may be evaluated in this article, or claim that may be made by its manufacturer, is not guaranteed or endorsed by the publisher.
